# Neuroprotective Effects of Licochalcone D in Oxidative-Stress-Induced Primitive Neural Stem Cells from Parkinson’s Disease Patient-Derived iPSCs

**DOI:** 10.3390/biomedicines11010228

**Published:** 2023-01-16

**Authors:** Minyoung Oh, Juhyeon Nam, Areum Baek, Ji-Hye Seo, Jung-Il Chae, Seo-Young Lee, Sun-Ku Chung, Byoung Chul Park, Sung Goo Park, Janghwan Kim, Young-Joo Jeon

**Affiliations:** 1 Stem Cell Convergence Research Center, Korea Research Institute of Bioscience and Biotechnology (KRIBB), Daejeon 34141, Republic of Korea; 2 Department of Functional Genomics, KRIBB School of Bioscience, University of Science and Technology, Daejeon 34113, Republic of Korea; 3 Department of Dental Pharmacology, School of Dentistry, BK21 Plus, Jeonbuk National University, Jeonju 54896, Republic of Korea; 4 Korean Medicine (KM) Science Research Division, Korea Institute of Oriental Medicine, Daejeon 34054, Republic of Korea; 5 Critical Diseases Diagnostics Convergence Research Center, Korea Research Institute of Bioscience and Biotechnology (KRIBB), Daejeon 34141, Republic of Korea; 6 Disease Target Structure Research Center, Korea Research Institute of Bioscience and Biotechnology (KRIBB), Daejeon 34141, Republic of Korea

**Keywords:** Parkinson’s disease, induced pluripotent stem cells, primitive neural stem cells, apotosis, Licochalcone D, JunD

## Abstract

Parkinson’s disease (PD) is one of the most common neurodegenerative diseases caused by the loss of dopaminergic neurons in the substantia nigra pars compacta. Although the etiology of PD is still unclear, the death of dopaminergic neurons during PD progression was revealed to be associated with abnormal aggregation of α-synuclein, elevation of oxidative stress, dysfunction of mitochondrial functions, and increased neuroinflammation. In this study, the effects of Licochalcone D (LCD) on MG132-induced neurotoxicity in primitive neural stem cells (pNSCs) derived from reprogrammed iPSCs were investigated. A cell viability assay showed that LCD had anti-apoptotic properties in MG132-induced oxidative-stressed pNSCs. It was confirmed that apoptosis was reduced in pNSCs treated with LCD through 7-AAD/Annexin Ⅴ staining and cleaved caspase3. These effects of LCD were mediated through an interaction with JunD and through the EGFR/AKT and JNK signaling pathways. These findings suggest that LCD could be a potential antioxidant reagent for preventing disease-related pathological phenotypes of PD.

## 1. Introduction

Parkinson’s disease (PD) is the second most common neurodegenerative disease after Alzheimer’s disease, having plagued humans for more than 200 years. The main pathological changes in PD are the formation of Lewy corpuscles and a decrease in dopaminergic neurons (DN) in the substantia nigra pars compacta (SNpc), which leads to a decrease in dopamine content in the related nerve endings and an imbalance between dopamine and acetylcholine [1,2,3]. The etiology of PD has not yet been fully understood. Although many possible pathogenetic mechanisms have been proposed over the years, including excessive release of oxygen free radicals during enzymatic dopamine breakdown, disruption of calcium homeostasis, impairment of mitochondrial function, loss of trophic support, abnormal kinase activity, dysfunction of protein degradation, and neuroinflammation, the pathogenesis of PD is still largely uncertain [4,5]. PD is recognized as a progressively complex neurodegenerative disease. It primarily affects the dorsal motor nucleus of the vagus nerve and the olfactory bulbs and nucleus, then the locus coeruleus, and eventually the SNpc. Cortical areas of the brain are affected at a later stage. Damage to these neuronal systems causes multifaceted pathophysiologic changes that cause damage not only to motor systems (resulting in tremor, postural disturbances, rigidity, or bradykinesia) but also to cognitive and neuropsychological systems (leading to sleep disorders, hyposmia, or neuropsychiatric, autonomic, and sensory symptoms). Moreover, deficits in control and motor inhibition [6,7]; motor imagery or suppression of ongoing action [8]; or emotion perception, reactivity, and regulation [9,10] that depend on abnormal neural activity in the prefrontal cortex (PFC) associated with severe impulsivity problems are characteristic features of neuropsychiatric disorders [11]. In addition, functional alterations in the PFC affect the memory and learning abilities of patients with psychiatric disorders and brain damage [12,13]. Symptoms worsen over time as increasingly more of the cells affected by the disease are lost. The course of the disease is highly variable, with some patients exhibiting very few symptoms as they age and others demonstrating rapidly progressive symptoms [14].

The majority of PD patients suffer from sporadic forms of the disease, and the familial form accounts for only approximately 10% of all cases [15,16]. Over the past few decades, several genes associated with PD, such as glucocerebrosidase, alpha-synuclein, and leucine-rich repeat kinase 2 (LRRK2), have been discovered through genome-wide association studies [17]. The most common mutation that causes PD is in LRRK2, which contains a combination of LRRK2 and is mainly associated with aberrant kinase activity [18]. All definite LRRK2 mutations are in the catalytic domains and may result in hyperactivation of the kinase domain [19]. LRRK2 is involved in a variety of cell biological processes, and the disease mechanisms may affect its critical role in microtubule function and Rab proteins as phosphorylation substrates [20]. An autosomal dominant genomic mutation (c.6055 G > A) results in LRRK2-p. The G2019S substitution is the most common genetic risk factor for PD [21]. In addition, it has been suggested that the LRRK2 G2019S (LK2GS) mutation reduces antioxidant defense mechanisms in mitochondria by increasing the levels of reactive oxygen species (ROS) [22]. Inhibition of oxidative stress through the induction of mitochondrial biogenesis using antioxidant reagents, showing protective effects on DN in PD models, could potentially be a promising therapeutic mediator against PD [23].

For PD research, the generation of model systems that accurately reflect LK2GS-associated disease states is particularly challenging. For example, animal models with the LK2GS mutation fail to show clear evidence of progressive dopaminergic neuron loss or Lewy body formation [24,25,26]. Another approach taken with PD models is the use of patient-derived induced pluripotent stem cells (iPSCs) directed to differentiate into dopamine neurons. However, since these cells are immature, they exhibit varying degrees of dopaminergic neurotoxicity, and other PD pathological features, including Lewy body aggregates, are not as prominent as in the human brain [27,28]. The primitive neural stem cells (pNSCs), although derived from iPSCs, are well-known in vitro models that better reflect the pathogenesis of PD than the above models. They can be cultured relatively constantly and reproducibly compared to conventional neural stem cells, which change from neurogenic to gliogenic within several passages [29,30,31].

Licochalcone D (LCD), a flavonoid isolated from the Chinese medicinal plant *Glycyrrhiza inflata*, is a relatively safe and effective natural product and has been reported to have anti-cancer, antioxidant, and anti-inflammatory effects [32,33]. In a previous study, LCD was used to facilitate the marked recovery of Langendorff-perfused rat models from myocardial ischemia injury [34]. Therefore, LCD treatment has the potential to be effective in alleviating neurodegenerative phenotypes in PD patients. However, animal models may not sufficiently represent the correlation between genetic factors, aging processes, and environmental damage in patients with PD [35]. Therefore, to evaluate the effect of LCD on PD, it is appropriate to use human-based models that represent the complex pathological phenotype of PD.

In this study, we simulated a PD model by treating primitive pNSCs reprogrammed from iPSCs with MG132, a proteasome inhibitor. We observed that apoptosis caused by MG132-induced oxidative stress was restored when LCD treatment was administered before the stress condition. These results confirm that LCD is involved in cell survival by regulating the EGFR/AKT and JNK signaling pathways. These findings suggest that LCD is a potential therapeutic agent for PD.

## 2. Materials and Methods

### 2.1. Cell Culture

Fibroblasts of a patient with PD harboring the LK2GS mutation (ND38262, ND14317) and healthy control subjects (AG02261) were purchased from the Coriell Institute (Camden, NJ, USA) and reprogrammed into iPSC [36,37]. In addition, ND14317 was corrected to generate the KIOMi002-A WT iPSC line to prepare an isogenic WT/G2019S pair [38]. iPSCs were differentiated into pNSCs and cultured as follows [31]. Healthy control-derived (WT) pNSCs and PD patient-derived (GS) pNSCs were plated on Geltrex™-coated plates with a pNSC medium: 50% Advanced DMEM/F12 (Thermo Fisher Scientific, Waltham, MA, USA), 50% Neurobasal medium (Thermo Fisher Scientific, Waltham, MA, USA), N-2 Supplement (100X, Thermo Fisher Scientific, Waltham, MA, USA), B-27 Supplement (50X, Thermo Fisher Scientific, Waltham, MA, USA), 2 mM GlutaMax™ (Thermo Fisher Scientific, Waltham, MA, USA), 1% penicillin/streptomycin (Thermo Fisher Scientific, Waltham, MA, USA), 3 uM CHIR 99021 (Tocris Bioscience, Bristol, UK), 2 uM SB 431542 (Tocris Bioscience, Bristol, UK), 10 ng/mL human LIF (PeproTech, Inc., Rocky Hill, NJ, USA), and 5 ug/mL BSA (Sigma-Aldrich, St. Louis, MO, USA). The medium was replaced every alternate day. WT- and LK2GS-pNSCs were dissociated with Accutase cell detachment solution (Millipore Sigma, Burlington, MA, USA) every five–seven days.

### 2.2. Cell Viability Assay

A total of 30,000 pNSCs/well were seeded in a Gletrex™-coated 96-well tissue culture microplate. The ROCK Inhibitor Y-27632 (Tocris Bioscience, Bristol, UK) was added to the pNSC medium at a concentration of 10 µM for 24 h. Various concentrations of LCD or equal volumes of DMSO were added to the pNSC medium for 24 h, followed by 10 µM MG132 (Sigma-Aldrich, St. Louis, MO, USA). Cell viability was measured using an EZ-cytoX-enhanced cell viability assay kit (DoGenBio, Seoul, Korea), according to the manufacturer’s instructions. After 24 h, absorbance was measured using a Spectramax microplate reader (Molecular Devices, San Jose, CA, USA).

### 2.3. Flow Cytometry Analysis

A total of 300,000 pNSCs/well were seeded in a Gletrex™-coated 12-well tissue culture microplate. The ROCK Inhibitor Y-27632 was added to the pNSC medium at a concentration of 10 µM for 24 h. LCD (2, 4 µM) or equal volumes of DMSO were added to the pNSCs medium for 24 h, followed by 10 µM MG132. For analysis, pNSCs were dissociated into single cells by incubation with Accutase cell detachment solution for 4 min. For apoptosis assays, the cells were stained with fluorescein Annexin V and 7-amino-actinomycin D (7-AAD, BD Pharmingen™, San Jose, CA, USA).

### 2.4. Western Blot Analysis

The cells were washed with DPBS and harvested with protein lysis buffer, composed of RIPA buffer (Sigma-Aldrich, St. Louis, MO, USA), Xpert protease inhibitor cocktail (100X, GenDEPOT, Katy, TX, USA), and PhosSTOP™ phosphatase inhibitor (10X, Roche, Basel, Switzerland). Total protein concentration was measured using the Pierce™ BCA Protein Assay Kit (Thermo Fisher Scientific, Waltham, MA, USA). Equal amounts of protein were boiled at 100 °C for 5 min and subjected to sodium dodecyl sulfate-polyacrylamide gel electrophoresis. Proteins were transferred onto a PVDF membrane (Bio-Rad, Hercules, CA, USA) using Wet/Tank Blotting Systems (Bio-Rad Laboratories, Inc., Hercules, CA, USA). The membrane was blocked with 5% skimmed milk (Difco Skim Milk, BD Biosciences, San Jose, CA, USA) in TBST (LPS solution, Daejeon, Korea) at room temperature for 30 min. The membranes were then incubated with primary antibodies (Table S1) overnight at 4 °C, followed by incubation with horseradish peroxidase (HRP)-conjugated secondary antibodies (Cell Signaling Technology, CST, Danvers, MA, USA). The ECL™ Select Western Blotting Detection Reagent (GE Healthcare, Chicago, IL, USA) was used to detect HRP signals. Protein band images were acquired using a LAS-3000 imaging system (Fujifilm, Minato, Tokyo, Japan).

### 2.5. Immunofluorescence

The cells were fixed in 4% paraformaldehyde for 10 min at room temperature and washed two to three times with DPBS. Then, the cells were blocked and permeabilized with 0.3% TritonX-100 (Sigma-Aldrich, St Louis, MO, USA) diluted in 3% bovine serum albumin (BSA; Thermo Fisher Scientific, Waltham, MA, USA) in DPBS for an hour at room temperature. The cells were incubated with primary antibodies (Table S1) in 1% BSA overnight at 4℃, followed by two to three washes with 0.1% BSA in DPBS. Next, the cells were incubated with the secondary antibodies (Thermo Fisher Scientific, Waltham, MA, USA) in 0.1% BSA for an hour at room temperature. The cells were washed two to three times and stained with Hoechst33342 (Thermo Fisher Scientific, Waltham, MA, USA) for 10 min at room temperature. Images were acquired using a microscope (EVOS M7000, Thermo Fisher Scientific, Waltham, MA, USA).

### 2.6. Quantitative Real-Time PCR

Total RNA was extracted from the cell pellets using a RNeasy Plus Mini Kit (QIAGEN, Hilden, Germany) according to the manufacturer’s recommendations [31]. First-strand cDNA was produced from 1 µg of total RNA using the iScript cDNA synthesis kit (Bio-Rad, Hercules, CA, USA), and qRT-PCR was performed using the 7500 Fast Real-Time PCR System (Applied Biosystems, Foster City, CA, USA).

### 2.7. In Vitro Pull-Down Assay

The following procedure has been described previously [39,40]. Briefly, LK2GS-pNSCs lysates were reacted with sepharose 4B beads or LCD-sepharose 4B beads in reaction buffer (50 mM Tris, pH 7.5; 5 mM EDTA; 150 mM NaCl; 1 mM dithiothreitol; 0.01% Nonidet P-40; 2 µg/mL bovine serum albumin; 0.02 mM phenylmethylsulfonyl fluoride; and 1X proteinase inhibitor) and washed five times with washing buffer (50 mM Tris, pH 7.5; 5 mM EDTA; 150 mM NaCl; 1 mM dithiothreitol; 0.01% Nonidet P-40; 0.02 mM phenylmethylsulfonyl fluoride). Proteins bound to the beads were analyzed by Western blot analysis using the JunD antibody.

### 2.8. Statistical Analysis

Results are presented as mean ± standard error of the mean of at least three independent experiments performed in triplicate. Statistical significance was assessed using a two-tailed Student’s *t*-test. *p*-values < 0.05 were considered statistically significant.

## 3. Results

### 3.1. Generation and Characterization of pNSCs from LK2GS Patients

**Figure 1:**
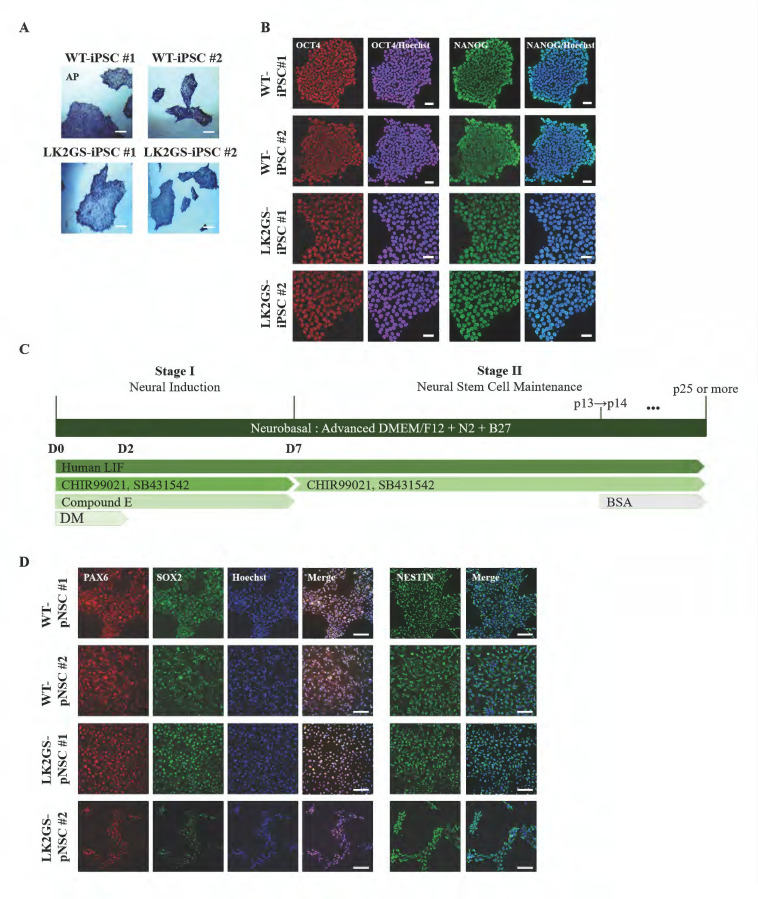
Generation of pNSCs using PD-patient-specific iPSCs. (**A**) AP staining of WT-iPSCs and LK2GS-iPSCs. Scale bars are 200 µm. (**B**) Immunostaining of pluripotent stem cell markers in WT- and LK2GS-iPSCs. Scale bars are 50 µm. (**C**) Schematic diagram of differentiation human iPSCs into neural stem cells. (**D**) Immunostaining of neural stem cell markers (PAX6, SOX2, NESTIN) in WT- and LK2GS-pNSCs. Scale bars are 125 µm.

In a previous study, we generated iPSCs from somatic cells of patients with PD harboring the LK2GS mutation (LK2GS-iPSC#1, LK2GS-iPSC#2) using non-integrating oriP/EBNA-1-based episomal vectors (Table 1). In addition, WT-iPSCs (WT-iPSC#1 and WT-iPSC#2) were generated using non-integrating oriP/EBNA-1-based episomal vectors and Sendai virus using normal somatic cells. We analyzed pluripotency by immunostaining for octamer-binding transcription factor 4 (OCT4) and NANOG as well as alkaline phosphatase reactivity (Figure 1A,B). In addition, the genomic stability analysis of WT- and LK2GS-iPSCs confirmed normal karyotypes (Figure S1) [31,38]. To generate pNSCs, we differentiated these iPSCs using a two-step protocol consisting of two steps: neural induction (stage 1) and pNSC maintenance (stage 2) (Figure 1C). We confirmed that these iPSCs expressed neural stem cell markers, such as paired box 6 (PAX6), SRY-box transcription factor 2 (SOX2), and nestin (NES) (Figure 1D). These results demonstrated the successful generation of WT- and LK2GS-iPSCs into differentiated pNSC.

### 3.2. LK2GS-pNSCs as an In Vitro Model for PD

**Figure 2:**
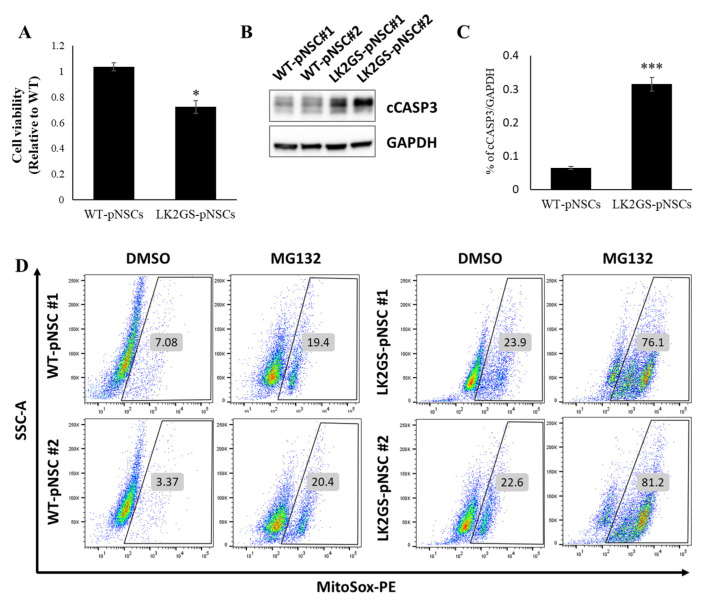
LK2GS-pNSCs as a PD model. (**A**) Cell viability assay with MG132 treatment. *p*-values were analyzed using the unpaired two-tailed Student’s *t*-test (* *p* < 0.05). (**B**) Representative Western blot for cleaved cCASP3 in WT- and LK2GS-pNSCs. GAPDH was used as an internal control. (**C**) Quantification of cCASP3 after normalization using GAPDH. *p*-values were analyzed using the unpaired two-tailed Student’s *t*-test (*** *p* < 0.001). (**D**) Mitochondrial reactive oxygen species (mtROS) of MG132-treated pNSCs stained with MitoSox.

Toxin-based protocols using different types of cellular stressors are being used to establish cellular models of PD [41]. Among them, MG132, a proteasome inhibitor, impairs the intracellular protein clearance system involved in the ubiquitin–proteasome system, leading to cell death [41,42,43]. Proteasome inhibitors have been widely used in PD modeling because PD-patient-derived cells exhibit more severe apoptosis induced by proteasome stress than healthy controls [41,42]. To determine whether our pNSCs represent a PD phenotype such as apoptosis, we treated them with MG132 or DMSO for 24 h. We examined whether MG132 could effectively inhibit the viability of LK2GS-pNSCs compared to that of WT-pNSCs. We observed more apoptotic cells in LK2GS-pNSCs than in WT-pNSCs after MG132 treatment (Figure 2A). In addition, we determined whether cleaved caspase3 (cCASP3) levels were altered by MG132 under the same conditions as in the viability assay. The level of cleaved caspase3 after MG132 treatment was dramatically increased in LK2GS-pNSCs compared to WT-pNSCs (Figure 2B,C). Furthermore, we investigated increased mitochondrial dysfunction after treatment with MG132 in LK2GS-pNSCs compared with that in WT-pNSCs (Figure 2D). These results suggest that pNSCs from patients with PD could be an appropriate in vitro model for PD through MG132 treatment.

### 3.3. LCD Attenuated MG132-Induced Cell Death of the PD Model

**Figure 3:**
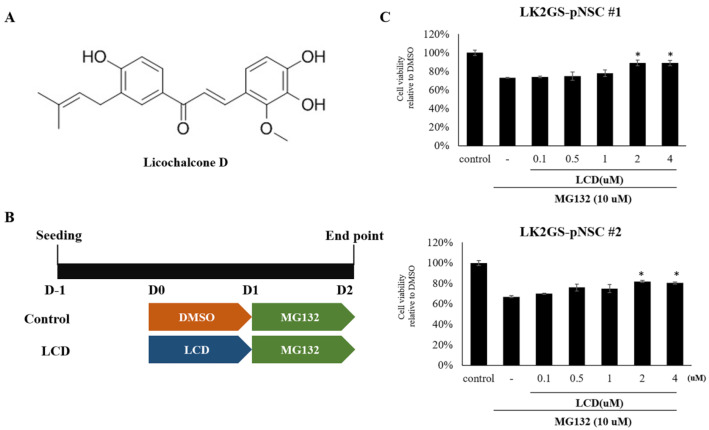
Cytoprotective effects of LCD on cellular stress in WT- and LK2GS-pNSCs. (**A**) Structure of LCD. (**B**) Schematic diagram of treatment of LCD and MG132 in WT- and LK2GS-pNSCs. (**C**) Cytoprotective effects of LCD (0.1~4 µM, 24 h) on WT- and LK2GS-pNSCs with the MG132 (10 µM, 24 h)-induced oxidative stress condition. Data are mean ± S.E.M of at least three independent experiments. *p*-values were analyzed using the unpaired two-tailed Student’s *t*-test (* *p* < 0.05).

To determine whether LCD (Figure 3A) exhibited a neuroprotective effect in our PD model, LK2GS-pNSCs were seeded in 96-well plates, cultured for 24 h, and treated with different concentrations of LCD for 24 h. Pre-conditioned cells were then treated with MG132 (Figure 3B). Viability analysis showed that MG132 decreased LK2GS-pNSC viability, while pretreatment with 2 and 4 µM LCD conferred significant protection against MG132-induced cell death (Figure 3C).

### 3.4. LCD Protects against MG132-Induced Apoptosis in the PD Model

**Figure 4:**
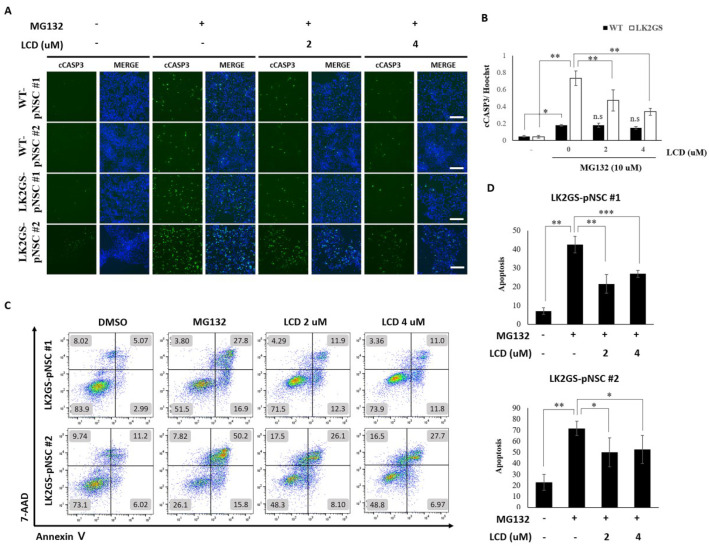
Effects of LCD on MG132-induced apoptosis in WT- and LK2GS-pNSCs. (**A**) Immunostaining of cCASP3 was used to evaluate MG132-induced apoptosis and recovery by LCD treatment. Scale bars are 125 µm. (**B**) Quantification of cCASP3 staining area after normalization using Hoechst staining area. *p*-values were analyzed using the unpaired two-tailed Student’s *t*-test (* *p* < 0.05, ** *p* < 0.01). (**C**) Flow cytometric analysis by Annexin V on the *x*-axis and 7-aminoactinomycin D concentration on the *y*-axis, following double staining of WT- and LK2GS-pNSCs treated with MG132 after LCD. (**D**) Quantitative data showing the percentage of apoptotic cells according to treatment (* *p* < 0.05, ** *p* < 0.01, *** *p* < 0.001).

To further evaluate the effectiveness of LCD in our in vitro PD model, cellular stress was induced by MG132 to mimic PD pathologies in WT and LK2GS-pNSCs following treatment with DMSO or LCD (2, 4 µM) for 24 h. We found that LK2GS-pNSCs showed significantly increased levels of cleaved caspase3 compared to WT-pNSCs under MG132-induced stress conditions. Furthermore, LCD-treated LK2GS-pNSCs exhibited significantly reduced levels of cleaved caspase3 within 24 h of treatment (Figure 4A,B). In addition, after treatment with MG132 and/or LCD for 24 h, the cells were stained with Annexin V and 7-AAD for flow cytometry. Cells are represented in a lot plot as health (Annexin-V^−^/7-AAD^−^), undergoing early apoptosis (Annexin-V^+^/7-AAD^−^), late apoptosis (Annexin-V^+^/7-AAD^+^) and necrosis (Annexin-V^−^/7-AAD^+^) quadrants (Figure 4C). LCD pretreatment reduced MG132-induced apoptosis in the PD model (Figure 4D). Therefore, we confirmed that MG132-induced apoptotic cell death in LK2GS-pNSCs was reduced by LCD.

### 3.5. LCD Administration Modulated Phosphorylation of EGFR/AKT and JNK in the MG132-Treated PD Model

**Figure 5:**
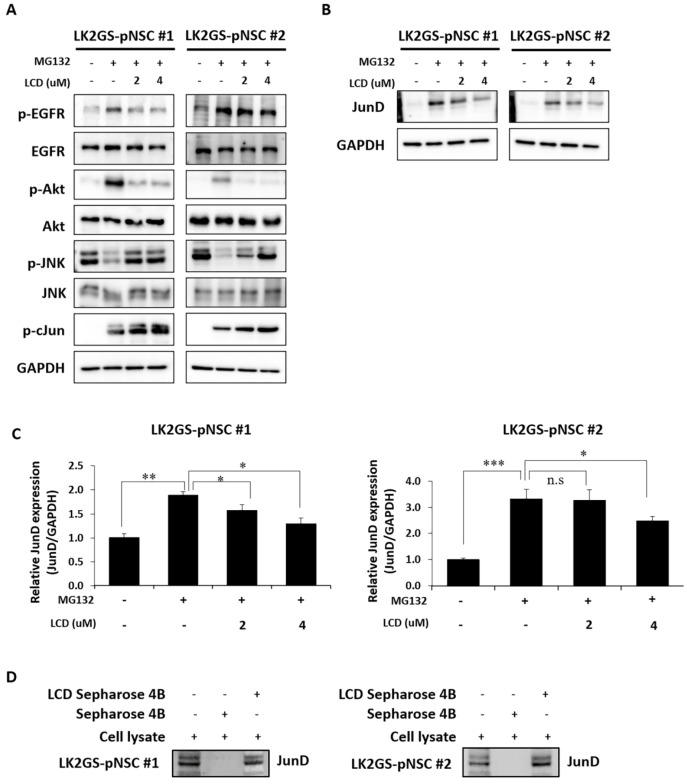
LCD administration regulates the phosphorylation of EGFR/AKT and JNK in MG132-treated with LK2GS-pNSCs. (**A**,**B**) Western blot analysis was performed with antibodies against p-EGFR, EGFR, p-AKT, AKT, p-JNK, JNK, p-cJun, and JunD. GAPDH was used as an internal standard protein. (**C**) qRT-PCR analyses showing relative expression levels of JunD transcripts. After normalization with GAPDH, results are means ± S.E.M for three independent experiments (* *p* < 0.05, ** *p* < 0.01, *** *p* < 0.001, n.s: no significance). (**D**) LCD binds JunD. Whole LK2GS-pNSCs lysates were incubated with Sepharose 4B or LCD-Sepharose 4B beads for 16 h at 4 °C. Beads were washed, and pulled-down JunD protein was visualized by Western blotting.

The EGFR/AKT and MAPK signaling pathways are involved in the regulation of cell survival and apoptosis after treatment with MG132 [44,45]. Thus, we evaluated the phosphorylation levels of p-EGFR, p-AKT, and MAPK in LK2GS-pNSCs after MG132 treatment using Western blotting (Figure 5A). EGFR phosphorylation levels increased in MG132-treated LK2GS-pNSCs but recovered to the level before MG132 administration in LCD-pretreated LK2GS-pNSCs. We further investigated whether MAPK, a downstream signal of EGFR, was affected by LCD treatment. Western blot analysis revealed that LCD treatment significantly activated JNK, which sequentially activated c-Jun in MG132-induced LK2GS-pNSCs (Figure 5A). LCD treatment did not affect the phosphorylation of ERK and p38. In addition, we focused on JunD, which is one of the components of activating protein-1(AP-1) among the subfactors of JNK [46]. As shown in Figure 5B,C, protein and mRNA levels of JunD were decreased by LCD in MG132-induced LK2GS-pNSCs. An in vitro pull-down assay was performed using LCD-conjugated Sepharose 4 B beads. JunD bound to LCD-conjugated Sepharose beads but not to Sepharose beads alone (Figure 5D). These results suggest that the LCD-mediated protective effect of apoptosis occurs via EGFR/AKT and JNK.

### 3.6. LCD Regulated the Expression of Apoptosis-Related Molecules in the PD Model

**Figure 6:**
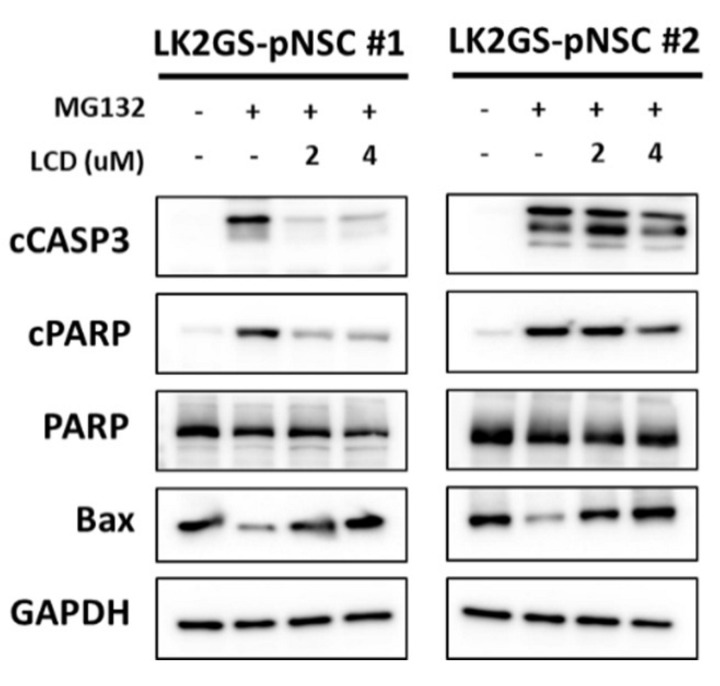
The effect of LCD on the expression of apoptosis-related proteins in MG132-induced LK2GS-pNSCs. The expressions of cCASP3, cPARP, PARP, and Bax were measured by Western blot. GAPDH was used as an internal standard protein.

The above data suggest that LCD regulated EGFR/AKT, and JNK regulated apoptosis. Therefore, we examined whether the treatment of cells with LCD regulated the expression levels of apoptosis-related proteins in MG132-induced apoptosis. As shown in Figure 6, LCD treatment attenuated the activation of caspase3 and PARP and increased the expression of Bax in MG132-induced LK2GS-pNSCs. Our results demonstrate that LCD alleviated EGFR/AKT and JNK, resulting in the recovery of apoptotic cell death in the PD model.

## 4. Discussion

The evidence for the occurrence of oxidative stress in PD is overwhelming. The central nervous system is very susceptible to ROS because it consumes large amounts of oxygen and is not particularly enriched in antioxidant defenses compared with other tissue [47]. LCD, an active flavonoid isolated from the Chinese medicinal herb Glycyrrhiza inflata, has antioxidative, anti-inflammatory, and anti-cancer activity [48]. According to previous studies, LCD showed protective effects against oxidative stress and myocardial injury [32,34]. Therefore, we hypothesized that LCDs might exhibit neuroprotective effects against oxidative stress in PD. One thing to consider in this study is the blood–brain barrier (BBB) permeability of LCD. Unfortunately, we have not directly checked whether the LCD passes through the BBB. However, the molecular weight of LCD is 354.4, and the number of hydrogen bonds is 3, which satisfies the conditions for passing the BBB [49]. Therefore, although there was a possibility that the LCD could pass through the BBB, we conducted the experiment. MG132, a cell-permeable proteasome inhibitor, has been used to establish a toxin-based protocol for PD modeling and has been used to induce mitochondrial dysfunction and ROS, ultimately promoting apoptosis in in vitro and in vivo PD [29,42,43].

PD is difficult to study because of a lack of experimental models, such as in vivo systems, and the complex perturbations of multiple biological processes. For this reason, it is important to use a model that well reflects the pathology of PD when studying possible protective effects against the disease. Our previous study demonstrated the potential of LK2GS-pNSCs with pathogenic features in patients with PD as an in vitro PD model system [31]. This in vitro PD model reflects the characteristics of PD etiology, such as oxidative stress. In this study, we evaluated the protective effect of LCD on LK2GS-pNSCs from patients with PD. LCD treatment was administered before the stress condition, and the pathological phenotypes of the cells were analyzed. Our in vitro PD model showed that 2–4 µM of LCD is suitable for protection against MG132-induced apoptosis (Figure 3 and Figure 4).

We detected the phosphorylation status of EGFR, AKT, and JNK after LCD pretreatment of cells treated with MG132, and Western blotting results showed that the phosphorylation of EGFR, AKT, and JNK was significantly regulated by LCD (Figure 5A). EGFR plays an essential role in cellular differentiation in the developing brain, which involves differentiation of both neurons and glial cells from neural stem cells, neural progenitor cells, and glial progenitor cells [50]. Under normal conditions, in the absence of injury or pathology, astrocytes exist as quiescent astrocytes with barely detectable levels of EGFR, but loss of normal astrocyte function is known to be associated with the pathology of neurodegenerative diseases [50,51,52,53]. Thus, knocking down EGFR or treatment with EGFR inhibitors can prevent oxidative-stress-induced neurodegeneration [54,55]. EGFR activity was increased in the condition treated with MG132, which mimics PD pathology, and was decreased by LCD. LCD exhibits a neuroprotective effect while acting as an inhibitor of EGFR. EGFR acts upstream of the PI3K/AKT/mToR pathway, which is associated with cell metabolism, proliferation, differentiation, and survival [56,57]. In the present study, the phosphorylated levels of EGFR/AKT pathways were rescued by LCD pretreatment in MG132-treated LK2GS-pNSCs.

JNK is a key regulator of a variety of cellular events and plays an important role in apoptosis, inflammation, and cell survival [58,59,60]. There is evidence that the balance between cell survival and apoptosis may be impaired during aging due to alterations in the MAPK cascades [61]. JNK is activated by upstream mixed-lineage kinases and may, therefore, play a role in LK2-associated kinase-mediated metabolism. In a previous study, JNK phosphorylation was reduced in patients with PD who had the LK2GS mutant compared to that in the control group [62,63]. In the present study, phosphorylated JNK was decreased in MG132-treated LK2GS-pNSCs compared to that in untreated LK2GS-pNSCs and recovered by LCD. Thus, the results proved that EGFR/AKT and JNK signaling are involved in protective mechanisms.

As JNK phosphorylation was dramatically regulated by LCD, we focused on the sub-factors of JNK. The AP-1 family of transcription factors are dimeric complexes composed of various Fos and Jun proteins. The three Jun proteins—cJun, JunD, and JunB—can either heterodimerize or homodimerize to form an AP-1 complex, and each show subtle but important differences in regulation and output [64]. In certain contexts, Jun proteins appear to play opposing roles. For example, in immortalized mouse embryonic fibroblasts, c-Jun promotes cell proliferation, whereas JunD inhibits it [65,66]. However, each Jun protein can influence cellular behavior in very different ways, depending on the context. For example, it promotes cell death in response to cellular stress or proliferation in response to growth factors [67,68,69]. Our results show that cJun phosphorylation is regulated by JNK signaling; however, the protein expression level of JunD showed the opposite trend. Our results showed that JunD protein and mRNA levels increased when cJun phosphorylation decreased in MG132-treated LK2GS-pNSCs. In contrast, JunD protein and mRNA levels decreased when p-cJun levels were increased by LCD. In addition, LCD directly binds to JunD. In our in vitro PD models, it is possible that the balance between cJun and JunD contributed to the variation in JNK signaling outcomes in these different contexts.

Although many breakthroughs in PD therapy have been accomplished, there is currently no cure for PD, only trials to alleviate the related motor symptoms [70]. Rather than providing symptomatic relief to reduce or halt clinical progression and mobility impairment, new drug therapies aimed at preventing the loss of dopamine-producing brain cells may be more effective in the early stages of PD.

In order to discuss the neuroprotective effect of LCD in PD patients, in-depth studies on the newly identified pathways and processes as well as confirmation in other models are needed. Additionally, if LCD plays a regulatory role in EGFR/AKT and JNK phosphorylation induced by MG132, further studies are needed to address which signaling pathways are mainly involved in protection from neurotoxicity.

## 5. Conclusions

In this study, we investigated the protective effect of LCD on pNSC derived from iPSCs of patients with LK2GS PD. LCD treatment restores oxidative-stress-induced apoptosis in LK2GS-pNSCs. In addition, LCD treatment significantly downregulated p-EGFR and p-AKT and upregulated p-JNK and cJun. Furthermore, LCD bound to JunD and reduced JunD expression levels in our in vitro PD model. The enhancement of these antioxidant molecules may lead to the attenuation of pathological phenotypes in LK2GS-pNSCs, an in vitro PD model. The present study suggests that LCD may be a neuroprotective agent that can be used to prevent neurodegenerative diseases.

## Figures and Tables

**Table 1:** List of cells used in this study.

	Cell Line	Reprogramming Method	Gender	Age
WT-pNSC #1	ND14317 cor	Episomal vector	Male	53
WT-pNSC #2	AG02261	Sendai virus	Male	61
LK2GS-pNSC #1	ND38262	Episomal vector	Male	60
LK2GS-pNSC #2	ND14317	Episomal vector	Male	53

## Data Availability

All data generated or analysed during this study are included in this published article.

## Supplementary Materials

The following supporting information can be downloaded at https://www.mdpi.com/article/10.3390/biomedicines11010228/s1, Figure S1: Karyotypes of the established WT- and LK2GS-iPSCs. Table S1: List of antibodies used in this study.

## Abbreviations

PD	Parkinson’s disease
SNpc	substantia nigra pars compacta
PFC	prefrontal cortex
LCD	Licochalcone D
iPSCs	induced pluripotent stem cells
pNSCs	primitive neural stem cells
LRRK2	leucine-rich repeat kinase 2
Lk2GS	LRRK2 G2019S
ROS	reactive oxygen species
PAX6	paired box 6
SOX2	SRY-box transcription factor 2
NES	Nestin
cCASP3	cleaved caspase-3
MAPK	mitogen-activated protein kinase
EGFR	epidermal growth factor receptor
AKT	serine/thereonine protein kinase B
JNK	c-Jun NH(2)-terminal kinases
PARP	poly (ADP-ribose) polymerase
PI3K	phosphoinositide 3-kinase
mTOR	mechanistic target of rapamycin
BBB	blood–brain barrier
