# Supplementary material for: Neuroprotective Effects of Licochalcone D in Oxidative-Stress-Induced Primitive Neural Stem Cells from Parkinson’s Disease Patient-Derived iPSCs
Source: Biomedicines. 2023 Jan 16;11(1):228. doi: 10.3390/biomedicines11010228 (PMC9856162; doi:10.3390/biomedicines11010228)
Supplement: Supplementary file 1 [file biomedicines-11-00228-s001.zip › biomedicines-2143293-supplementary.pdf]

Table S1 List of the antibodies used for ICC and Western blotting.

Immunofluorescence

Antibody	Supplier	Product No.	Dilution
OCT4	Santa Cruz	sc-9081	1:500
NANOG	R&D Systems	AF1997	1:40
PAX6	BioLegend	901301	1:500
SOX2	Millipore	MAB4343	1:200
NESTIN	Millipore	MAB5326	1:200
Cleaved Caspase3	Cell Signaling Technology (C.S.T)	9661	1:400

Western blot

Antibody	Supplier	Product No.	Dilution
p-EGFR	C.S.T	3777S	1:1000
EGFR	Santa Cruz	sc-71033	1:1000
p-SAPK/JNK	C.S.T	9251S	1:1000
JUND	Santa Cruz	sc-271938	1:1000
p-AKT	C.S.T	4060	1:1000
AKT	C.S.T	9272	1:1000
p-c-Jun	C.S.T	3270	1:1000
Cleaved Caspase3	C.S.T	9661	1:1000
Cleaved PARP (Asp214)	C.S.T	9541	1:1000
PARP	Sigma-Aldrich	p7605	1:1000
Bax	C.S.T	41162	1:1000
GAPDH	Santa Cruz	sc-47724	1:3000
